# Temperature-Driven Maillard Conjugation and Phenolic Changes in Dried Lychee Pulp: Implications for Antioxidative Enhancement

**DOI:** 10.3390/foods15030468

**Published:** 2026-01-29

**Authors:** Supakit Chaipoot, Kanokwan Kulprachakarn, Pairote Wiriyacharee, Chalermkwan Somjai, Kuntathee Chaimueng, Sirinthip Jaijoi, Apinya Khampakool, Worachai Wongwatcharayothin, Sirasit Srinuanpan, Pattavara Pathomrungsiyounggul, Rewat Phongphisutthinant

**Affiliations:** 1 Multidisciplinary Research Institute, Chiang Mai University, Chiang Mai 50200, Thailand; kuntathee.c@gmail.com (K.C.); ninesrt1991@gmail.com (S.J.); 2 Center of Excellence in Microbial Diversity and Sustainable Utilization, Chiang Mai University, Chiang Mai 50200, Thailand; pairote.w@cmu.ac.th (P.W.); sirasit.s@cmu.ac.th (S.S.); 3 School of Health Sciences Research, Research Institute for Health Sciences, Chiang Mai University, Chiang Mai 50200, Thailand; kanokwan.kul@cmu.ac.th; 4 Processing and Product Development Factory, The Royal Project Foundation, Chiang Mai 50100, Thailand; chalermkwansomjai@hotmail.com; 5 Faculty of Humanity, Chiang Mai University, Chiang Mai 50200, Thailand; apinya.nanattapin@gmail.com (A.K.); worachai.w@cmu.ac.th (W.W.); 6 Department of Biology, Faculty of Science, Chiang Mai University, Chiang Mai 50200, Thailand; 7 Faculty of Agro-Industry, Chiang Mai University, Chiang Mai 50100, Thailand; pattavara.p@cmu.ac.th

**Keywords:** thermal aging processing, Maillard reaction, antioxidant activity, bioactive compounds, natural antioxidant ingredient

## Abstract

Thermal aging is an effective strategy for improving the functional properties of fruit-based ingredients via physicochemical modifications. This research investigates the effect of thermal aging on the physicochemical, antioxidant, and Maillard conjugation properties of dried lychee pulp aged at 50, 60, 70, and 80 °C for 20 days under controlled relative humidity. Comprehensive analyses were performed, including total phenolic content (TPC), total flavonoid content (TFC), individual phenolic profiles, saccharide composition, free amino acid content, degree of glycation (DG), peptide molecular weight distribution, and antioxidant activity assessed by ABTS, DPPH, and FRAP assays. The results demonstrated that aging at moderate temperatures (60–70 °C) significantly enhanced TPC, TFC, and antioxidant activity, alongside an increased degree of glycation, peaking at approximately 47% at 70 °C. Principal component analysis (PCA) confirmed strong correlations between these compositional changes and antioxidant responses. In contrast, aging at 80 °C led to the degradation of thermolabile phenolics, sugars, and amino acids, resulting in reduced antioxidant activity compared with non-aged samples. Overall, the results highlight a temperature-dependent balance between constructive Maillard conjugation and thermal degradation, identifying 60–70 °C as an optimal aging range for improving the functional quality of dried lychee pulp. These findings provide mechanistic insight into thermal modulation of fruit bioactivity and support the potential application of controlled thermal aging in the development of value-added functional food ingredients.

## 1. Introduction

The lychee cultivar *Litchi chinensis* Sonn. var. Hong Huay is one of approximately 20 commercially valuable varieties, with the majority cultivated in northern Thailand, and the species originates in China [1,2,3]. Lychee is a tropical fruit capable of thriving in both tropical and subtropical climates [4,5,6,7]. In the food industry, lychee pulp is widely processed into value-added products such as canned fruit, dried pulp, frozen desserts, beverages, wine, syrup, and jelly [8]. This versatility is attributed to its distinctive aroma, sweet flavor, and high nutritional content. Often referred to as the “king of fruits” in China, lychee has been used in traditional practices for thousands of years and is also associated with various modern pharmacological health benefits [9]. Lychee offers multiple health benefits because it is rich in bioactive compounds, especially flavonoids and other phenolics. Phenolic compounds from lychee pulp have been shown to improve gut health by enhancing intestinal barrier integrity, modulating gut microbiota, and activating anti-inflammatory pathways, thereby offering protection against colitis [10]. Lychee is also an important source of diverse bioactive constituents, including polyphenols (e.g., flavonoids, phenolic acids, and flavan-3-ols), as well as terpenes, steroids, and minerals. These compounds have attracted increasing interest in applications for food, cosmetics, and pharmaceutical products. Moreover, gallic acid, catechin, epicatechin, procyanidin, malvidin, quercetin, and rutin isolated from lychee have demonstrated antioxidant, anticancer, antidiabetic, anti-obesity, and anti-inflammatory activities in both cell-based and animal studies [11]. The fruit also shows hepatoprotective effects by reducing oxidative stress and apoptosis in liver injury models due to its vitamin C and phenolic content [12,13]. The lychee pulp is a rich source of sugars, vitamins, and minerals. Fresh lychee pulp contains approximately 82–84% water, 15–17% carbohydrates, 0.1–0.8% protein, and 0.4–0.7% fat. It also provides notable amounts of vitamin C (about 10–15 mg/100 g) and phosphorus (approximately 32 mg/100 g), contributing significantly to its overall nutritional value [5,6].

Drying is a practical and commercially scalable method for extending the shelf life of fragile fruits such as lychee. A comparative study by Huang et al. [14] revealed that drying lychee pulp modifies its polysaccharide composition and enhances bioactivity. Similarly, Somjai et al. [15] reported that prolonged thermal aging of dried longan pulp enhanced antioxidant activity and cholinesterase inhibitory effects. These enhancements have been attributed to the Maillard reaction, which involves interactions between reducing sugars and amino groups, resulting in the formation of a wide range of bioactive compounds [16,17]. This reaction is increasingly recognized as a strategy to improve the nutraceutical value of fruit or plant-based ingredients by generating compounds with antioxidant, anti-inflammatory, and functional properties [18,19,20,21]. Consequently, thermal aging strategies have attracted considerable attention, with improvements in antioxidant capacity, phenolic availability, and biofunctional properties in products such as dried longan [15,22], dried shiitake mushroom [16], dried sloe berries [20], black orange [23], and black garlic [24]. Additionally, research on lychee pulp drying techniques, including air drying, heat pump drying, infrared drying, freeze drying, and vacuum freeze drying, shows that thermal conditions directly affect their biological properties [25]. Interestingly, maintaining 75% relative humidity preservation of postharvest fruits helps slow respiration and senescence, thereby extending shelf life and reducing shriveling and browning [26]. Lychee pulp dried by a hot-air oven to a final moisture content of 23–26% (wet basis) exhibits textural and flavor characteristics comparable to those of dried raisins [4,27]. However, there are currently no studies examining the effects of different aging temperatures on dried lychee pulp over a fixed incubation period, particularly in relation to Maillard reactions involving reducing sugars and amino groups.

Therefore, the present study aimed to examine the effects of aging at different temperatures (50, 60, 70, and 80 °C) on dried lychee pulp under controlled incubation conditions. The study evaluated changes in phenolic acid profiles, physicochemical properties, and antioxidant activity before and after aging, as these factors may be linked to Maillard conjugation. Furthermore, correlations among physical, chemical, and antioxidant parameters were examined to better understand the impact of thermal aging. It was hypothesized that increasing the aging temperature would promote Maillard reaction progression, leading to structural and compositional changes that enhance antioxidant properties up to an optimal point before thermal degradation dominates.

## 2. Materials and Methods

### 2.1. Materials and Chemicals

Lychee (*Litchi chinensis* Sonn. var. Hong Huay) was sourced from Chiang Mai Province, Thailand, during its cultivation period in April–May 2024. The chemical substances, reagents, and standards applied in this research included 3,5-dinitrosalicylic acid (DNS) 98% (Sigma-Aldrich, Bangalore, India), 2,2-diphenyl-1-picrylhydrazyl (DPPH) (Sigma-Aldrich, India), Trolox (Sigma-Aldrich, Buchs, Switzerland), 2,4,6-tris(2-pyridyl)-s- triazine (TPTZ) (Sigma-Aldrich, Switzerland), gallic acid 99% (Sigma-Aldrich, St. Louis, MO, USA), *O*-phthaldialdehyde (OPA) (Sigma-Aldrich, Shanghai, China), detached phenol crystals 99.5% (Loba Chemie, Mumbai, India), ferric chloride hexahydrate (FeCl_3_ 6H_2_O) (Loba Chemie, India), potassium persulfate (Loba Chemie, India), di-sodium tetraborate decahydrate (Borax) AR/ACS (Loba Chemie, India), sodium dodecyl sulfate (SDS) (Loba Chemie, India), sodium hypochlorite (Loba Chemie, India), boric acid (Loba Chemie, India), sodium acetate trihydrate (KemAus, Sydney, Australia), ferrous sulfate heptahydrate (FeSO_4_ 7H_2_O) (QReC, Auckland, New Zealand), 2,2′-azino-bis(3-ethylbenzothiazoline-6-sulfonic acid) (ABTS) (Sigma-Aldrich, China), sodium carbonate (Na_2_CO_3_) (KemAus, Australia), D-glucose (Unilab, Springvale, Australia), Folin–Ciocalteu’s phenol (Merck, Darmstadt, Germany), 2-mercaptoethanol (Merck, Germany), N-acetyl-L-cysteine (Merck, Germany), L-lysine monohydrochloride (HiMedia, Mumbai, India), glacial acetic acid, acetonitrile HPLC, ethanol AR, hexane AR, methanol AR, hydrochloric acid 37% AR, sulfuric acid 98%, potassium sulfate, sodium citrate tribasic dihydrate, and sodium hydroxide were purchased from RCI Labscan, Bangkok, Thailand. A set of 17 analytical amino acids was purchased from Wako Pure Chem. Co., Tokyo, Japan. A water purification system (Zeneer UP 900, Seoul, Republic of Korea) was used.

### 2.2. Lychee Sample Preparation

The method for preparing the lychee samples followed the method moist–dry–heating system described by Somjai et al. [22], with some modifications. Whole fresh lychee fruits were dried using a hot-air dryer (Model UNE 600, Memmert, Schwabach, Germany) at 60 °C until the moisture content reached 16–18% to obtain dried lychee pulp, which served as the control sample (non–aged). Subsequently, 300 g of dried lychee pulp was incubated in desiccators maintained at 75% relative humidity using a saturated NaCl solution and placed in an incubator (Daihan Scientific, Seoul, Republic of Korea) at varying temperatures (50 °C, 60 °C, 70 °C, and 80 °C) for 20 days. The aged samples (Lyc_50 °C, Lyc_60 °C, Lyc_70 °C, and Lyc_80 °C) were then sealed in polyethylene bags and stored at −18 °C for further analysis.

### 2.3. Dried Lychee Pulp Extraction for Chemical and Antioxidant Analysis

The process of lychee pulp extraction followed the method described by Somjai et al. [22], with some modifications. Dried lychee pulp (dehulled; 100 g) was mixed with distilled water at a 1:5 (*w*/*v*) ratio and blended using a hand mixer (800 W, Philips, Bangkok, Thailand) for 2 min. The resulting homogenate was subjected to ultrasonic extraction for 10 min at 25 °C, followed by centrifugation at 8000 rpm for 15 min to separate the soluble fraction. The supernatant was then filtered through Whatman No. 4 filter paper (pore size 20–25 μm) and subsequently lyophilized to obtain a concentrated lychee extract. The extract was stored in amber bottles at −18 °C until further analysis.

### 2.4. Assessment of Color, Moisture, Water Activity, and pH in Lychee Pulp Samples

Color measurements of the lychee pulp samples were conducted using a colorimeter (CR-400, Konica Minolta, Tokyo, Japan), calibrated with a standard white plate under D65 illumination (Y = 85.8, x = 0.314, y = 0.331). The results were expressed in the CIE Lab system, where L denotes lightness (0 = black, 100 = white), a* represents the red–green axis (positive = red, negative = green), and b* indicates the yellow–blue axis (positive = yellow, negative = blue) [28]. The total color difference (ΔE) throughout the aging period at each temperature was calculated in comparison to the control sample (dried lychee pulp before aging). The ΔE value was determined using Equation (1).

(1) ∆E = [(∆L*)^2^ + (∆a*)^2^ + (∆b*)^2^]^0.5^

Moisture content was determined according to the AOAC standard method [29]. Water activity (aw) was measured at 25 °C using a three-channel water activity meter (Novasina, Pfäffikon, Switzerland). For pH analysis, 10 g of the lychee pulp sample was mixed with 10 mL of distilled water and stirred for 5 min, followed by homogenization using a hand mixer for 30 s. The pH of the resulting solution was then measured using a pH meter (pHTestr30, Thermo Scientific Eutech, Singapore).

### 2.5. Chemical Composition Analysis of Lychee Pulp Extracts

#### 2.5.1. Determination of Phenolic Profiles by HPLC and Quantification of Total Phenolic and Flavonoid Contents by Spectrophotometry

**Figure 1:**
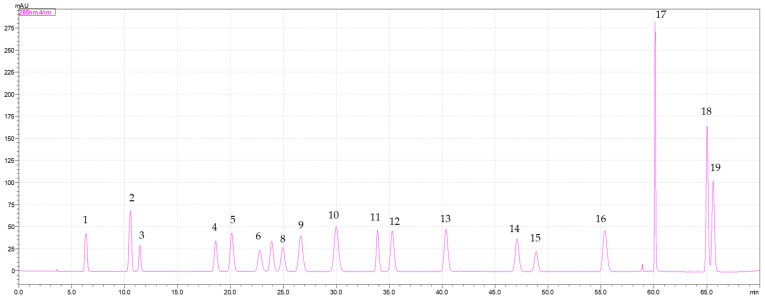
The chromatogram of nineteen phenolic standard compounds, including (1) gallic acid, (2) theobromine, (3) protocatechuic acid, (4) *p*-hydroxybenzoic acid, (5) catechin, (6) chlorogenic acid, (7) caffeine, (8) vanillic acid, (9) caffeic acid, (10) syringic acid, (11) epicatechin, (12) vanillin, (13) *p*-coumaric acid, (14) ferulic acid, (15) sinapic acid, (16) rutin, (17) myricetin, (18) quercetin, and (19) trans-cinnamic acid.

All lychee pulp samples (control sample, aged samples at 50, 60, 70, and 80 °C) were analyzed using an HPLC system (Shimadzu, Kyoto, Japan) equipped with a Prominence Diode Array Detector (DAD) and a C18 column (250 × 4.6 mm, GL Sciences, Torrance, CA, USA). The analysis employed a binary gradient system consisting of mobile phase A (2% acetic acid in water) and mobile phase B (100% acetonitrile), operated at a flow rate of 1 mL/min over a total run time of 85 min. The column oven was maintained at 30 °C. The solvent gradient was applied as described by Chaipoot et al. [16]. For sample preparation, 500 µL of each lychee extract was mixed with 500 µL of absolute acetonitrile to obtain a 2× diluted extract. The mixture was vortexed and then filtered through a 0.45 µm membrane filter to obtain a clear solution. An injection volume of 10 µL was used for analysis at 280 nm. A mixed standard containing 19 phenolic compounds was prepared, including gallic acid, theobromine, protocatechuic acid, *p*-hydroxybenzoic acid, catechin, chlorogenic acid, caffeine, vanillic acid, caffeic acid, syringic acid, epicatechin, vanillin, *p*-coumaric acid, ferulic acid, sinapic acid, rutin, myricetin, quercetin, and trans-cinnamic acid. Nineteen authentic phenolic compounds were used for identification and quantification by HPLC. The chromatogram of the phenolic standards is shown in Figure 1.

The lychee extract samples were diluted 5-fold with deionized water and subsequently used for both TPC and TFC analyses, following the method described by Zhang et al. [30]. TPC was expressed as milligrams of gallic acid equivalents (GAE) per 100 g of extract on a dry weight basis, while TFC was expressed as milligrams of quercetin equivalents (QE) per 100 g of extract on a dry weight basis.

#### 2.5.2. Analysis of Reducing Sugars, Mono- and Di-Saccharides

Reducing sugar content was quantified using a spectrophotometric colorimetric method following the protocol of Gandhi et al. [31]. Mono- and disaccharide contents were analyzed using high-performance liquid chromatography (HPLC), as described by Somjai et al. [22]. All analyses were conducted on extracts prepared from dried lychee pulp samples, and the results were expressed on a dry weight basis (db).

#### 2.5.3. Analysis of Available Free Amino Acids and Degree of Glycation (DG) Using UV-Vis Spectrophotometry

According to Somjai et al. [22], *O*-phthaldialdehyde (OPA) was used to quantify free amino groups in the aged lychee extract samples by measuring absorbance at 340 nm using a UV-vis spectrophotometer. Results were expressed as milligrams of lysine per 100 g of dry weight. The degree of glycation (DG) was calculated as the percentage decrease in absorbance relative to the control sample, reflecting the extent of conjugation at different aging temperatures (50, 60, 70, and 80 °C).

#### 2.5.4. Post-Column Amino Acid Analysis

A mixed standard solution containing 17 amino acids, namely alanine (Ala), arginine (Arg), aspartic acid (Asp), cystine (Cys), glutamic acid (Glu), glycine (Gly), histidine (His), isoleucine (Ile), leucine (Leu), lysine (Lys), methionine (Met), phenylalanine (Phe), proline (Pro), serine (Ser), threonine (Thr), tyrosine (Tyr), and valine (Val), was used for analysis via post-column fluorescence derivatization. *O*-phthalaldehyde (OPA) and N-acetylcysteine served as derivatizing agents for detecting amino acids hydrolyzed from proteins under sodium-based (Na-type) hydrolysis conditions [16,22].

#### 2.5.5. Peptide Size Distribution by Size-Exclusion Chromatography

The molecular weight distribution of soluble peptides in lychee extracts was analyzed by SEC using an SRT-C SEC-300 column (5 μm, 7.8 × 300 mm; Sepax Technologies, Inc., Newark, DE, USA) equipped with a DAD set at 280 nm, following the method describe by Phongphisutthinant et al. [17]. Separation was performed at 30 °C under isocratic conditions with 0.1 M sodium phosphate buffer (pH 7.0) at a flow rate of 0.1 mL/min for 20 min, using an injection volume of 10 µL. A protein standard mixture was used to estimate molecular weight distribution, including bovine thyroglobulin (~670 kDa), gamma globulins from bovine blood (150 kDa), albumin from chicken egg (grade VI; 44.3 kDa), ribonuclease A type I-A from bovine pancreas (13.7 kDa), and a low-molecular-weight marker, *p*-aminobenzoic acid (pABA). These standards covered a molecular weight range of approximately 15 to 600 kDa. A calibration curve was generated by plotting the logarithm of molecular weights against their corresponding elution times. The relative molecular size distribution of the lychee extract samples was calculated based on the area under the chromatographic curve, grouped into three peptide size ranges: <100 kDa, 101–1000 kDa, and >1000 kDa.

### 2.6. Evaluation of Antioxidant Activity Using FRAP, ABTS, and DPPH Assays

Antioxidant activity was evaluated using the FRAP and ABTS assays, which are based on electron transfer mechanisms, and the DPPH assay, which involves a combined electron and hydrogen atom transfer mechanism. The antioxidant capacities of lychee extracts were expressed as follows: FRAP results in mg FeSO_4_, ABTS results in mg GAE, and DPPH results in mg Trolox equivalent (TE), all per 100 g dry basis of the extract [16,17].

### 2.7. Statistical Analysis

Statistical analysis was conducted using SPSS software (version 17.0; SPSS Inc., Chicago, IL, USA). One-way ANOVA followed by Tukey’s post hoc test was used to determine significant differences, with a threshold of *p* ≤ 0.05. Additionally, a principal component analysis (PCA) biplot was generated using SPSS to visualize the correlations between aged lychee treatments at different temperatures and various parameters, including phenolic content, amino acids, conjugated properties, sugar profiles, physical characteristics, and antioxidant capacity. The analyses were performed in triplicate.

## 3. Results

### 3.1. Physical Qualities of Dried Lychee Pulps Aged at Different Temperatures

**Figure 2:**
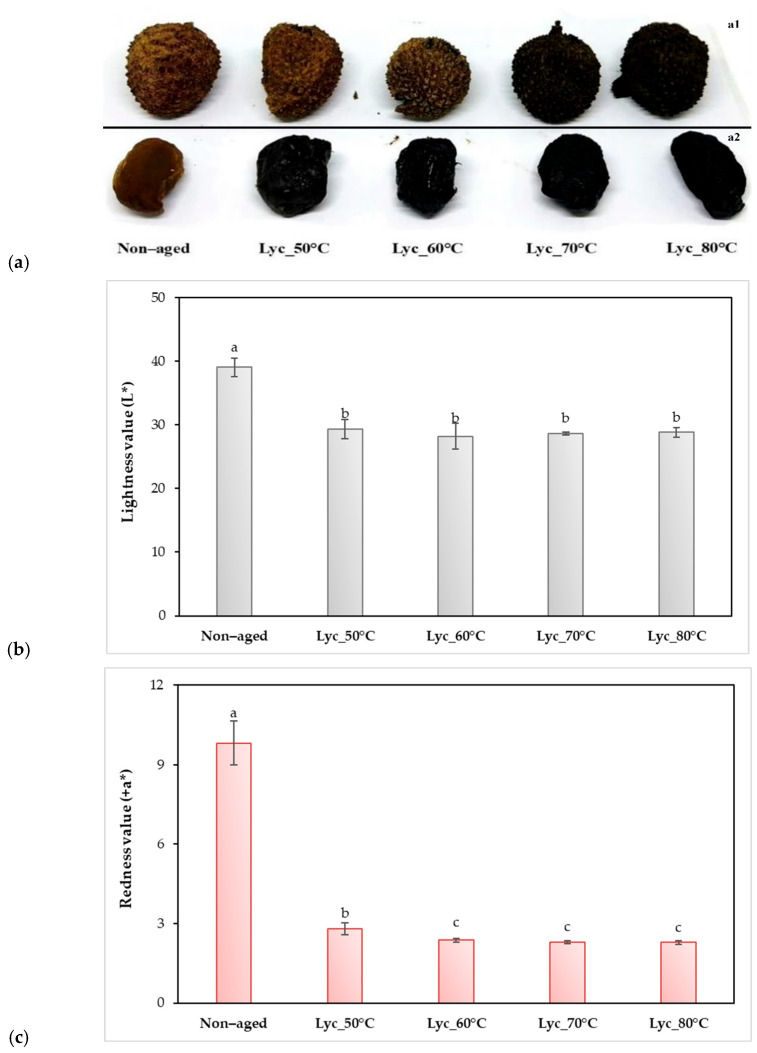

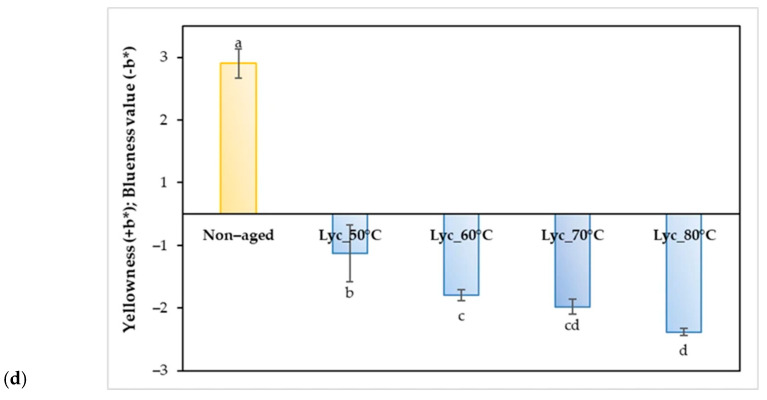
Lychee pulp samples: (**a**) visual appearance after 20 days of aging at 50 °C, 60 °C, 70 °C, and 80 °C compared to the non-aged control with whole fruit (**a1**) and dehulled (**a2**); (**b**–**d**) corresponding color parameters: (**b**) lightness (L*), (**c**) redness (+a*), and (**d**) yellowness (+b*) and blueness (−b*). Results are expressed as mean ± SD (n = 3), and significant differences among treatments (*p* ≤ 0.05) were indicated by different letters.

The appearance of both lychee peels and pulps darkened progressively after the aging process, developing a brownish hue relative to the non-aged samples shown in Figure 2a. Colorimetric measurements revealed a decrease in lightness (L*), redness (a*), and blueness (−b*) values with increasing aging temperature, relative to the non-aged control. However, no significant differences in L* values were observed among the aged treatments. In contrast, a* and −b* values were highest in samples aged at temperatures exceeding 60 °C. The color parameter ranges were L* 28.23–39.05, a* 2.28–9.82, and b* −1.88 to 2.40, as shown in Figure 2b–d. The total color difference (ΔE) ranged from 12.35 to 13.69, with no significant differences observed among the aged samples, as shown in Table 1. Thermal aging initially increased moisture content and water activity, with maximum values at 50 °C, followed by a gradual decline at temperatures above 70 °C, while non-aged samples consistently exhibited the lowest moisture levels. Increasing aging temperature significantly reduced pH, indicating enhanced acidity in lychee peels and pulps, which is associated with Maillard reaction progression and polyphenol oxidation intensified by heat and aging duration. The formation of acidic Maillard intermediates and oxidative byproducts during prolonged heating further contributed to pH reduction [32,33]. During early aging at 50 °C and 75% RH, moisture is rapidly absorbed from humid air via Fickian diffusion and then stabilizes at equilibrium [34], whereas at higher temperatures or longer aging times, water is consumed by chemical reactions and volatile products are formed, leading to decreases in both moisture content and pH [35].

### 3.2. Phenolic Profiles, Total Phenolic Content, and Total Flavonoid Content of Dried Lychee Pulps Aged at Different Temperatures

Phenolic compounds, including both phenolic acids and flavonoids, were evaluated in all dried lychee pulp extract samples to investigate the effect of thermal aging. The concentrations of individual phenolic compounds exhibited varied trends in response to elevated aging temperatures. HPLC analysis of dried lychee pulp using 19 phenolic standards showed total quantified phenolic contents ranging from 0.02 to 10.47 mg/100 g db (Table 2). The increase in total phenolic content observed at 60 °C was associated with higher levels of major phenolic compounds, including gallic acid, protocatechuic acid, chlorogenic acid, caffeic acid, and vanillic acid, as well as the appearance of catechin and epicatechin at moderate aging temperatures (50–70 °C). In contrast, the decline at 80 °C corresponded to the degradation of thermolabile phenolics, particularly catechin, epicatechin, chlorogenic acid, and quercetin, indicating that excessive thermal aging reduces antioxidant capacity through the loss of key phenolic compounds. This result relates to the excessive heat accelerating the thermal degradation of phenolics during high temperature [36,37]. Additionally, *p*-hydroxybenzoic acid was detected only after aging at 80 °C, suggesting its possible formation as a degradation product of other phenolic structures during high-temperature treatment. Heat treatment enhances total phenolic and flavonoid contents by decomposing macromolecular polyphenol complexes into smaller, bioavailable molecules while reducing the formation of esterified and glycosylated derivatives [23,38].

**Figure 3:**
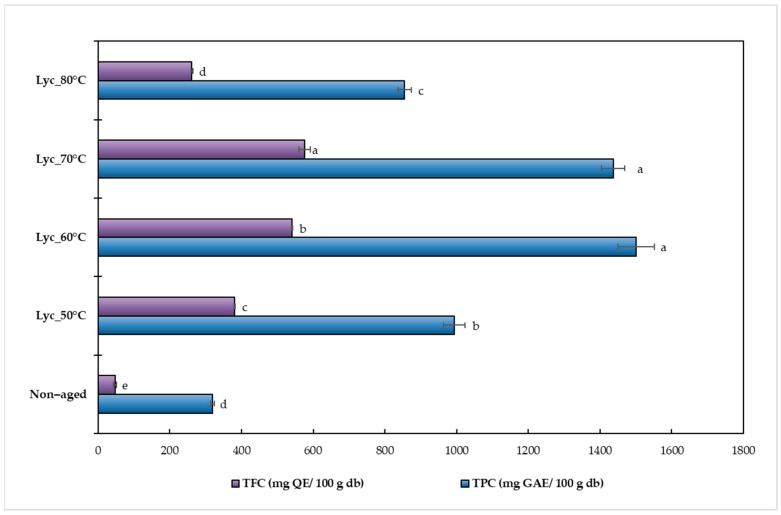
Total phenolic content (TPC) and total flavonoid content (TFC) of dried lychee samples aged at different temperatures for 20 days. Results are expressed as mean ± SD (n = 3), and significant differences among treatments (*p* ≤ 0.05) are indicated by different letters.

The total phenolic content (TPC) and total flavonoid content (TFC) of dried lychee samples aged at different temperatures for 20 days were also evaluated to confirm the effect of the aging process. The TPC and TFC levels ranged from 319 to 1500 mg GAE/100 g db and 47 to 577 mg quercetin equivalent (QE)/100 g db, respectively, as shown in Figure 3. Both TPC and TFC showed significant increases compared to the non-aged samples (*p* ≤ 0.05). The highest TPC was found at 60 °C, approximately 1500 ± 50 mg GAE/100 g db, and the highest TFC was found at 70 °C, approximately 576 ± 15 mg quercetin equivalent (QE)/100 g db. Notably, the TPC determined by the Folin–Ciocalteu assay reflects the combined contribution of all phenolic compounds present in the sample, including those not individually identified or quantified by HPLC. Therefore, the total phenolic content of the 19 phenolic compounds is expected to be lower than the TPC value obtained using the Folin–Ciocalteu method. These results align with the findings of Stephenus et al. [39], who reported that drying fruit at 50–60 °C enhances extraction yield. Similarly, the present study showed that aging at 50–70 °C significantly increases both TPC and TFC. This enhancement is attributed to thermal softening of plant tissues, which disrupts cellular structures and weakens the interactions between phenolic compounds and macromolecules (e.g., proteins and polysaccharides), thereby facilitating the release and diffusion of polyphenols into the extraction solvent [40]. However, a marked decline in both TPC and TFC was observed at 80 °C. This reduction result agrees with the findings of Che Sulaiman et al. [41], who reported that extraction efficiency decreases at 80 °C due to the thermal degradation of heat-sensitive bioactive compounds. As expected, the non-aged samples exhibited significantly lower TPC and TFC values compared with all thermally aged samples, confirming the moderate heat treatment in enhancing phenolic extractability.

### 3.3. Saccharide Compounds: Reducing Sugars, Monosaccharides, and Disaccharides in Dried Lychee Pulps Aged at Different Temperatures

Lychee fruit contains a high sugar content, with fructose, glucose, and sucrose as the predominant sugars. Saccharide compositions of dried lychee pulp samples aged at different temperatures for 20 days are presented in Table 3. The highest reducing sugar content was observed in samples aged at 50 °C (82.16 g/100 g db), followed by the non-aged control (79.82 g/100 g db). With increasing aging temperature, reducing sugar levels decreased at 60 °C (66.85 g/100 g db), 70 °C (49.27 g/100 g db), and 80 °C (42.50 g/100 g db), primarily due to their consumption in Maillard reactions at elevated temperatures. Similarly, glucose and fructose contents decreased with increasing temperature [33,42,43], while sucrose gradually declined and became undetectable at 80 °C, indicating thermal degradation and participation in non-enzymatic browning reactions [44,45]. In contrast, mannose and the rare sugar allulose increased with aging temperature, suggesting their formation through sugar isomerization and epimerization of fructose [15,46]. Overall, prolonged aging and higher temperatures reduced reducing sugars through accelerated Maillard reactions and concurrent sugar structural transformations.

### 3.4. Conjugation-Related Properties (Available Free Amino Acids, Degree of Glycation, Amino Acid Profiles, and Peptide Molecular Weight Distribution) of Dried Lychee Pulps Aged at Different Temperatures

**Figure 4:**
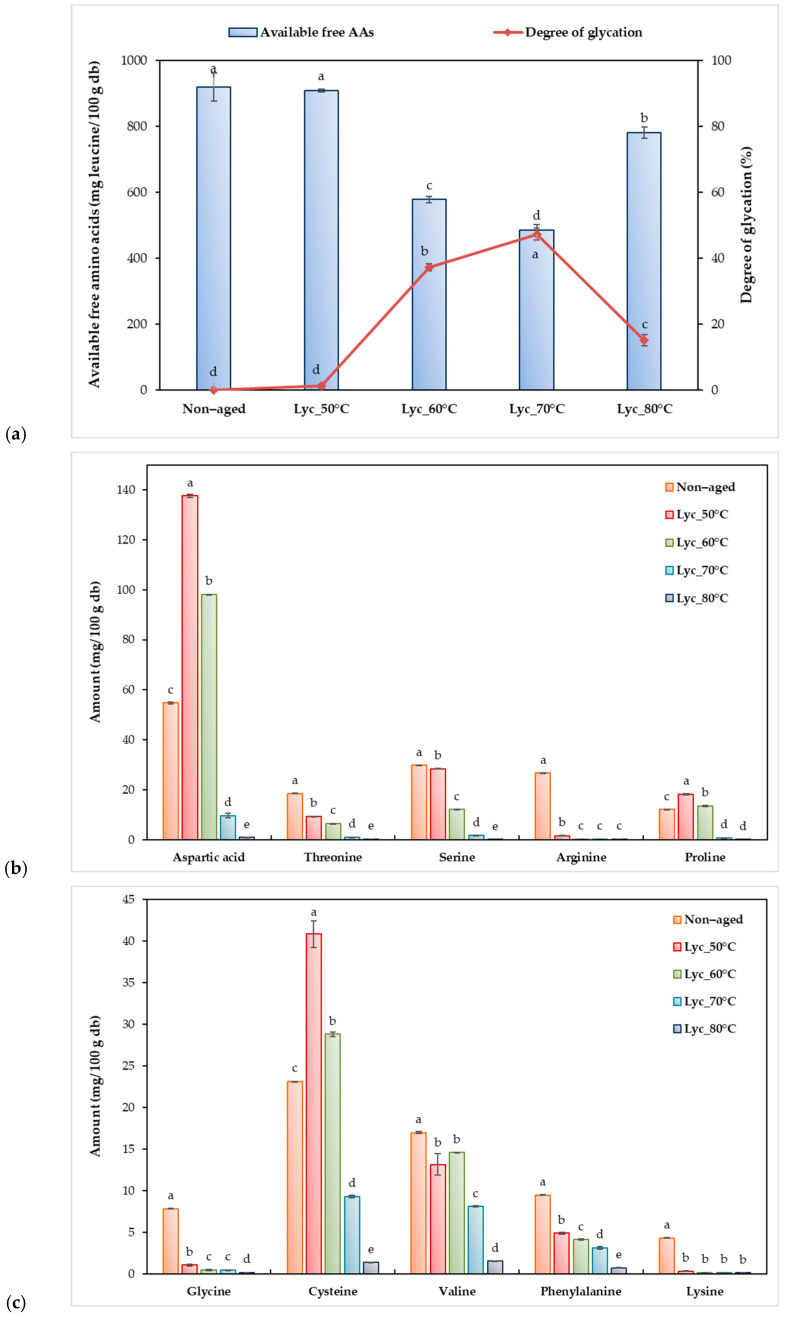

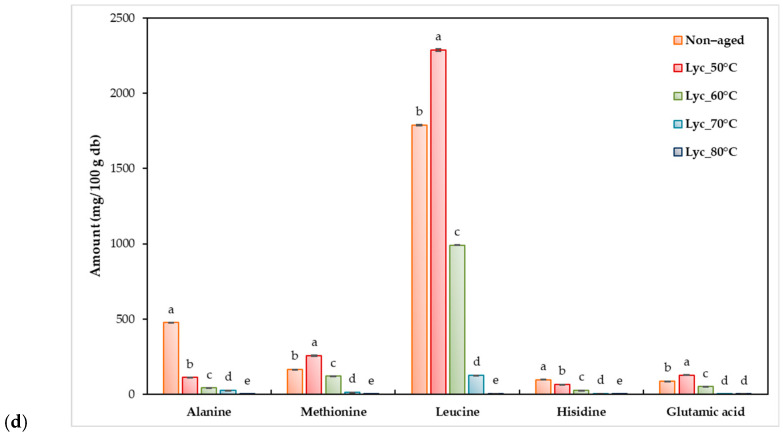
(**a**) Quantification of available free amino acids and the degree of glycation in dried lychee samples aged at different temperatures for 20 days; (**b**–**d**) Profiles of selected individual amino acids: (**b**) aspartic acid, threonine, serine, arginine, and proline; (**c**) glycine, cysteine, valine, phenylalanine, and lysine; (**d**) alanine, methionine, leucine, histidine, and glutamic acid. Results are expressed as mean ± SD (n = 3). Significant differences among treatments (*p* ≤ 0.05) are indicated by different letters.

**Figure 5:**
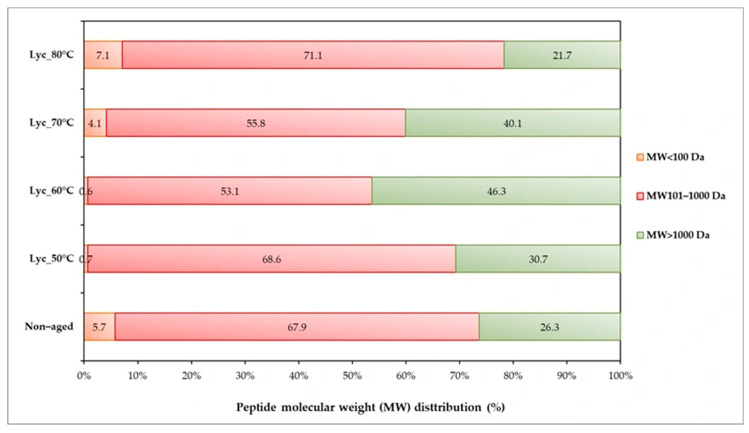
Distribution of peptide molecular weight analyzed by HPLC in dried lychee samples aged at different temperatures for 20 days.

To examine Maillard conjugation between amino acids and reducing sugars, free amino acid levels and the degree of glycation were analyzed. Dried lychee pulp aged at 70 °C showed the highest glycation degree (~47%) and lowest free amino acid content (~485 mg leucine/100 g db), indicating substantial amino acid consumption, as shown in Figure 4a. This result represents the formation of the Maillard reaction, indicated by an increase in glycation and a decrease in free amino acid content [47]. A similar trend was observed at 60 °C, where the degree of glycation reached 37%. In contrast, the samples aged at 50 °C and the non-aged controls exhibited the highest levels of free amino acids and the lowest glycation degrees, with no significant difference between them. Furthermore, excessive heat treatment at 80 °C showed degradation of Maillard conjugates, as indicated by an increase in free amino acid levels compared to those at 70 °C. To further investigate these changes, individual amino acids were quantified using HPLC, as shown in Figure 4b–d. The amino acid profile of dried lychee pulp showed noticeable patterns before and after thermal aging. The control (non-aged) dried lychee pulp contained high concentrations of six amino acids, namely leucine, alanine, methionine, histidine, glutamic acid, and aspartic acid (54.78–1787.60 mg/100 g db), while nine amino acids were present at lower concentrations (<50 mg/100 g db), namely serine, arginine, cysteine, threonine, proline, valine, phenylalanine, glycine, and lysine. Isoleucine and tyrosine were below detection limits in all samples. After aging, thermal aging induced temperature-dependent changes in amino acid composition. Six amino acid concentrations, namely leucine, methionine, cysteine, aspartic acid, proline, and glutamic acid, increased to maximum levels at 50 °C before declining at temperatures ≥ 60 °C. Meanwhile, all remaining detectable amino acids showed a progressive decrease with increasing aging temperature. The distribution of peptide molecular weights in lychee pulp extracts aged at different temperatures is shown in Figure 5. Peptides were classified into three molecular weight (MW) ranges: <100 Da, 101–1000 Da, and >1000 Da. The non-aged control already contained a substantial proportion of >1000 Da components (26.3%), indicating the presence of intrinsic high-molecular-weight peptides or proteins in the original material. Aging at moderate temperatures increased the proportion of >1000 Da peptides, reaching maximum values at 60 °C (46.3%) and 70 °C (40.1%), suggesting enhanced peptide aggregation and Maillard-reaction–related crosslinking. In contrast, aging at 80 °C reduced the >1000 Da fraction (21.7%) and increased the proportion of <100 Da peptides (7.1%), indicating peptide degradation under excessive thermal conditions. Peptides in the 101–1000 Da range were present across all samples (53.1–71.1%), reflecting dynamic peptide transformation during aging [17,22,48].

### 3.5. Antioxidant Activity and Its Correlation with Physicochemical and Conjugation-Related Properties of Dried Lychee Pulps Aged at Different Temperatures

**Figure 6:**
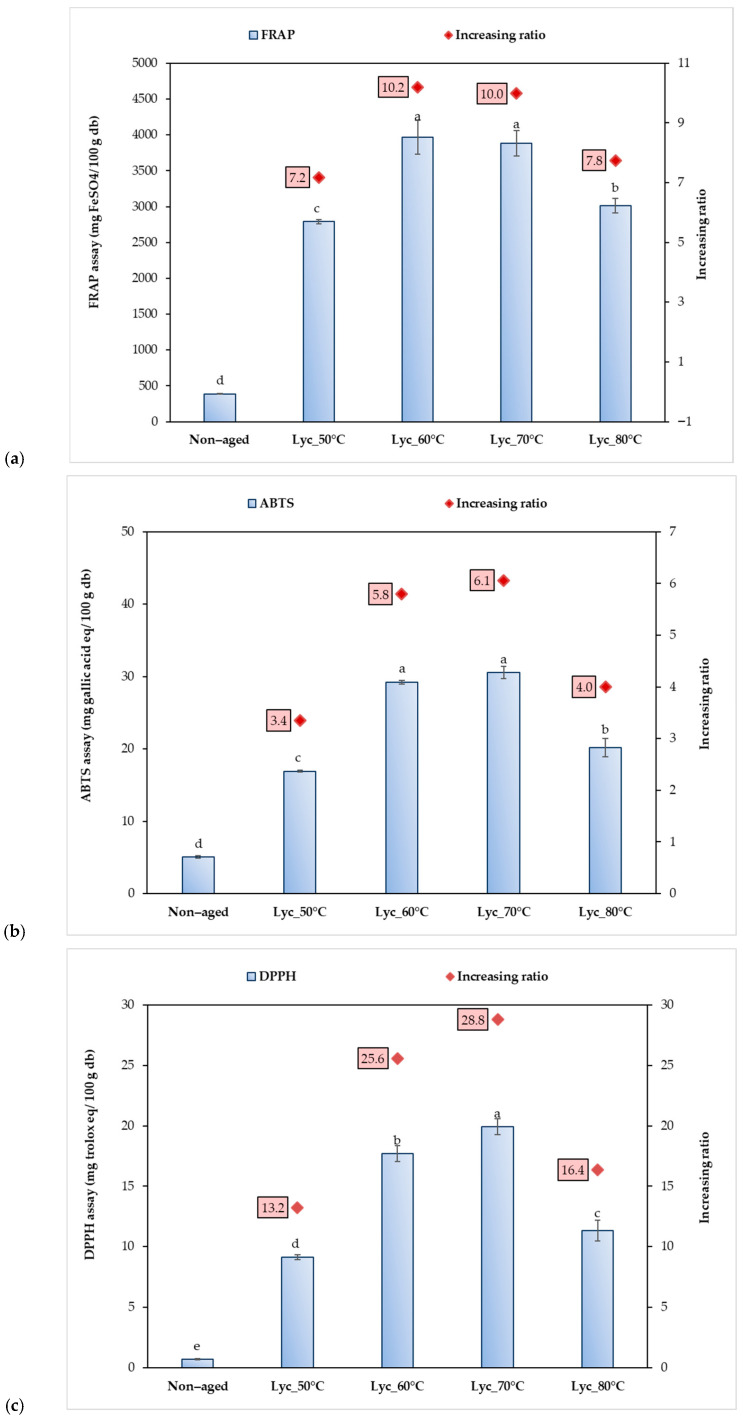
Antioxidant activity and relative increases in antioxidant capacity of lychee samples aged at different temperatures for 20 days, evaluated by (**a**) ABTS, (**b**) DPPH, and (**c**) FRAP assays, compared to the non-aged control. Results are expressed as mean ± SD (n = 3). Different letters indicate statistically significant differences among treatments (*p* ≤ 0.05).

**Figure 7:**
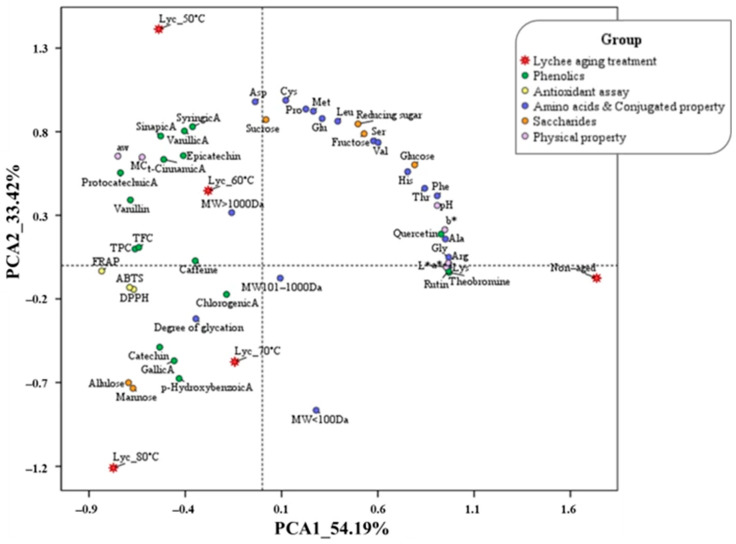
Principal component analysis (PCA) biplot showing PC1 (54.19%) and PC2 (33.42%), illustrating the correlation among physicochemical properties and antioxidant activity of lychee samples aged at different temperatures after 20 days.

All lychee extracts exhibited significantly higher antioxidant activities after the aging process compared to the non-aged control across all assays (FRAP, ABTS, and DPPH), as shown in Figure 6a–c. Although samples aged at 80 °C showed lower antioxidant capacity than those aged at 50–70 °C, their activities remained significantly higher than the non-aged sample. The results show that FRAP values ranged from 388.7 to 3012.8 mg FeSO_4_/100 g db, ABTS radical scavenging activity ranged from 5.0 to 30.6 mg GAE/100 g db, and DPPH radical scavenging activity ranged from 0.7 to 19.9 mgTE/100 g db. Among the aging treatments, samples aged at 70 °C exhibited the greatest enhancement in antioxidant capacity, with DPPH activity increasing by approximately 29-fold relative to the non-aged control, followed by FRAP (10-fold) and ABTS (6-fold). These results indicate that moderate aging temperature (70 °C) effectively enhances antioxidant activity, while excessive heat (80 °C) leads to partial degradation of antioxidant compounds, particularly phenolic acids, resulting in reduced antioxidant activity compared to the non-aged sample [37,49,50]. In addition, principal component analysis (PCA) was conducted to evaluate differences among lychee pulp samples aged at various temperatures, considering physicochemical properties, saccharides, amino acids, phenolic compounds, antioxidant activities, and Maillard reaction–related characteristics of dried lychee pulp, as shown in Figure 7. The first two principal components (PC1 and PC2) explained a total of 87.61% of the variance, with PC1 accounting for 54.19% and PC2 for 33.42%, indicating a strong representation of the dataset. Along PC1, the non-aged sample was clearly separated on the positive side, showing strong associations with reducing sugars (glucose, fructose, sucrose), free amino acids, and several low-molecular-weight compounds, reflecting minimal progression of thermal-induced reactions. In contrast, lychee samples subjected to thermal aging (50–80 °C) were distributed toward the negative side of PC1, indicating pronounced compositional and functional changes induced by aging. Moderate aging temperatures (50–70 °C) were closely associated with increased total phenolic content (TPC), total flavonoid content (TFC), antioxidant activities (DPPH, ABTS, FRAP), and a higher degree of glycation. These samples also showed strong correlations with phenolic acids and flavonoids such as gallic acid, catechin, chlorogenic acid, vanillic acid, and *p*-hydroxybenzoic acid, suggesting enhanced extractability and accumulation of bioactive compounds as well as the formation of Maillard-derived conjugates. Additionally, aging at 60–70 °C was related to intermediate molecular-weight fractions (101–1000 Da), indicating progressive conjugation between amino acids and sugars. Conversely, the sample aged at 80 °C was positioned distinctly along PC2 and negatively associated with most antioxidant and phenolic variables, suggesting thermal degradation of heat-sensitive compounds at excessive temperatures. This sample was also weakly correlated with sugar derivatives such as allulose and mannose, reflecting advanced thermal transformations. Overall, the PCA results demonstrate that controlled aging at moderate temperatures (60–70 °C) promotes favorable physicochemical and functional modifications, while excessive heat treatment (80 °C) leads to nutrient degradation and diminished bioactive potential. These multivariate relationships might confirm that phenolic accumulation and Maillard reaction–related transformations are the dominant contributors to quality enhancement during thermal aging of dried lychee pulp.

## 4. Conclusions

This study demonstrates that thermal aging influences the physicochemical properties, antioxidant capacity, and Maillard reaction–related characteristics of dried lychee pulp. Moderate aging temperatures (60–70 °C) create favorable conditions for controlled Maillard reactions, promoting conjugation between amino acids and reducing sugars while preserving thermolabile phenolics and flavonoids. This condition enhances antioxidant capacity by increasing phenolic availability and generating Maillard-derived conjugates with redox activity. In contrast, excessive thermal treatment at 80 °C, accelerates sugar consumption, amino acid degradation, and phenolic breakdown, which reduce antioxidant potential. These findings highlight temperature-dependent competition between Maillard conjugation and thermal degradation, identifying 60–70 °C as an optimal aging temperature for lychee pulp aged for 20 days. The findings provide valuable mechanistic insight into the thermal modulation of fruit bioactivity; further cellular and in vivo studies are required to confirm biological relevance, safety, and applicability before industrial implementation.

## Figures and Tables

**Table 1:** Total color difference (ΔE), moisture content, water activity, and pH of dried lychee samples aged at different temperatures for 20 days.

Aging Temperature (°C)	Total Color Difference (ΔE) ^ns^	Moisture Content (%)	Water Activity (aw)	pH
Non–aged	-	17.65 ± 0.82 ^d^	0.55 ± 0.02 ^d^	4.03 ± 0.02 ^a^
50	12.35 ± 0.45	28.85 ± 1.06 ^a^	0.70 ± 0.01 ^a^	3.52 ± 0.03 ^b^
60	13.69 ± 0.09	27.95 ± 0.68 ^ab^	0.65 ± 0.01 ^b^	3.49 ± 0.01 ^b^
70	13.41 ± 0.12	25.52 ± 0.61 ^b^	0.62 ± 0.01 ^bc^	3.28 ± 0.02 ^c^
80	13.40 ± 0.06	20.37 ± 0.72 ^c^	0.61 ± 0.01 ^c^	3.21 ± 0.02 ^d^

^a–d^ Different letters within the same column indicate significant differences among aging temperatures (*p* ≤ 0.05); ^ns^ = not significant (*p* > 0.05). Data are presented as mean ± SD (n = 3).

**Table 2:** Phenolic profiles of dried lychee samples aged at different temperatures for 20 days.

Phenolics (mg/100 g db)	Non–Aged	Aging Temperature (°C)			
		50	60	70	80
Gallic acid	1.11 ± 0.02 ^c^	1.34 ± 0.02 ^b^	1.22 ± 0.03 ^b^	2.15 ± 0.19 ^a^	1.73 ± 0.15 ^ab^
Theobromine	0.58 ± 0.02 ^a^	ND	ND	ND	ND
Protocatechuic acid	1.52 ± 0.08 ^d^	10.47 ± 0.08 ^a^	8.79 ± 0.26 ^b^	8.21 ± 0.05 ^b^	4.97 ± 0.21 ^c^
*p*-Hydroxybenzoic acid	ND	ND	ND	ND	1.67 ± 0.01 ^a^
Catechin	ND	0.59 ± 0.02 ^c^	0.50 ± 0.08 ^c^	2.02 ± 0.06 ^a^	1.16 ± 0.03 ^b^
Chlorogenic acid	0.21 ± 0.05 ^d^	0.71 ± 0.02 ^c^	2.12 ± 0.04 ^b^	5.34 ± 0.03 ^a^	0.29 ± 0.03 ^d^
Caffeine	0.56 ± 0.01 ^d^	0.70 ± 0.01 ^b^	0.81 ± 0.04 ^a^b	0.98 ± 0.02 ^a^	0.60 ± 0.02 ^c^
Vanillic acid	0.09 ± 0.01 ^c^	1.35 ± 0.01 ^a^	1.32 ± 0.04 ^a^	0.72 ± 0.15 ^b^	ND
Caffeic acid	ND	ND	ND	ND	ND
Syringic acid	0.07 ± 0.01 ^d^	0.54 ± 0.03 ^a^	0.41 ± 0.04 ^b^	0.32 ± 0.04 ^c^	ND
Epicatechin	0.07 ± 0.01 ^c^	1.15 ± 0.02 ^a^	1.10 ± 0.03 ^ab^	1.02 ± 0.10 ^b^	ND
Vanillin	0.02 ± 0.01 ^d^	0.85 ± 0.01 ^b^	1.13 ± 0.02 ^a^	0.87 ± 0.03 ^b^	0.43 ± 0.01 ^c^
*p*-Coumaric acid	ND	ND	ND	ND	ND
Ferulic acid	ND	ND	ND	ND	ND
Sinapic acid	0.07 ± 0.03 ^d^	0.57 ± 0.01 ^a^	0.56 ± 0.02 ^a^	0.28 ± 0.01 ^b^	0.14 ± 0.02 ^c^
Rutin	0.90 ± 0.02 ^a^	0.06 ± 0.01 ^b^	0.07 ± 0.02 ^b^	0.04 ± 0.01 ^c^	0.06 ± 0.02 ^b^
Myricetin	ND	ND	ND	ND	ND
Quercetin	0.19 ± 0.01 ^a^	0.03 ± 0.01 ^c^	0.07 ± 0.02 ^b^	ND	ND
trans-Cinnamic acid	0.02 ± 0.02 ^c^	0.07 ± 0.03 ^ab^	0.09 ± 0.02 ^a^	0.05 ± 0.01 ^b^	0.03 ± 0.01 ^c^

^a–d^ Different letters within the same column indicate significant differences among aging temperatures (*p* ≤ 0.05). ND = not detected; db = dry weight basis. Data are presented as mean ± SD (n = 3).

**Table 3:** Saccharide compositions of dried lychee pulp samples aged at different temperatures for 20 days.

Sugar Contents (g/100 g db)	Non–Aged	Aging Temperature (°C)			
		50	60	70	80
Reducing sugar	79.82 ± 0.28 ^b^	82.16 ± 0.16 ^a^	66.85 ± 0.64 ^c^	49.27 ± 0.31 ^d^	42.57 ± 0.58 ^e^
Glucose	22.38 ± 0.06 ^a^	18.33 ± 0.06 ^b^	16.01 ± 0.20 ^c^	13.33 ± 0.02 ^d^	10.53 ± 0.24 ^e^
Fructose	29.36 ± 0.32 ^a^	28.95 ± 0.30 ^a^	27.15 ± 0.37 ^ab^	24.05 ± 0.13 ^b^	13.36 ± 0.65 ^c^
Sucrose	0.33 ± 0.03 ^a^	0.32 ± 0.01 ^a^	0.22 ± 0.01 ^b^	0.18 ± 0.01 ^c^	ND
Mannose	ND	0.29 ± 0.01 ^d^	0.72 ± 0.01 ^c^	1.23 ± 0.03 ^b^	1.66 ± 0.04 ^a^
Allulose	ND	0.16 ± 0.01 ^c^	0.36 ± 0.02 ^b^	0.45 ± 0.01 ^b^	0.89 ± 0.03 ^a^

^a–d^ Different letters within the same column indicate significant differences among aging temperatures (*p* ≤ 0.05). ND = not detected; db = dry weight basis. Data are presented as mean ± SD (n = 3).

## Data Availability

The original contributions presented in the study are included in the article; further inquiries can be directed to the corresponding author.

## References

[1] Subhadrabandhu S., Yapwattanaphun C. (2001). lychee and longan production in thailand. Acta Hortic..

[2] Wiriya-Alongkorn W., Rasananda S. (2010). Thai tropical lychees—Identification of cultivars by morphological method. Acta Hortic..

[3] Charoenkit N., Naphrom D., Sruamsiri P., Sringarm K., Fukuda S. (2015). Modeling the Relationship between Hormone Dynamics and Off-season Flowering of Litchi by Using Random Forests. Agric. Agric. Sci. Procedia.

[4] Sivakumar D., Korsten L. (2011). Litchi (*Litchi chinensis* Sonn.). Postharvest Biology and Technology of Tropical and Subtropical Fruits: Cocona to Mango.

[5] Pareek S. (2016). Chapter 17—Nutritional and Biochemical Composition of Lychee (*Litchi chinensis* Sonn.) Cultivars. Nutritional Composition of Fruit Cultivars.

[6] Emanuele S., Lauricella M., Calvaruso G., D’Anneo A., Giuliano M. (2017). *Litchi chinensis* as a Functional Food and a Source of Antitumor Compounds: An Overview and a Description of Biochemical Pathways. Nutrients.

[7] Bano A., Singh U.P. (2024). A Review on Pharmacognosy, Phytochemistry and Pharmacological Properties of *Lychee chinensis*. Int. J. Pharm. Sci. Med..

[8] Yao P., Gao Y., Simal-Gandara J., Farag M.A., Chen W., Yao D., Delmas D., Chen Z., Liu K., Hu H. (2021). Litchi (*Litchi chinensis* Sonn.)*: A* comprehensive review of phytochemistry, medicinal properties, and product development. Food Funct..

[9] Sun W., Shahrajabian M.H., Shen H., Cheng Q. (2021). Lychee (*Litchi chinensis* Sonn.), the King of Fruits, with Both Traditional and Modern Pharmacological Health Benefits. Pharmacogn. Commun..

[10] Huang G., Wang Z., Wu G., Zhang R., Dong L., Huang F., Zhang M., Su D. (2021). Lychee (*Litchi chinensis* Sonn.) Pulp Phenolics Activate the Short-Chain Fatty Acid-Free Fatty Acid Receptor Anti-inflammatory Pathway by Regulating Microbiota and Mitigate Intestinal Barrier Damage in Dextran Sulfate Sodium-Induced Colitis in Mice. J. Agric. Food Chem..

[11] Castillo-Olvera G., Sandoval-Cortes J., Ascacio-Valdes J.A., Wong-Paz J.E., Álvarez-Pérez O.B., FloresLópez M.L., Aguilar C.N. (2025). *Litchi chinensis*: Nutritional, functional, and nutraceutical properties. Food Prod. Process. Nutr..

[12] Bhoopat L., Srichairatanakool S., Kanjanapothi D., Taesotikul T., Thananchai H., Bhoopat T. (2011). Hepatoprotective effects of lychee (*Litchi chinensis* Sonn.): A combination of antioxidant and anti-apoptotic activities. J. Ethnopharmacol..

[13] Queiroz E., Abreu C., Rocha D., Sousa R., Fráguas R., Braga M., César P. (2017). Lychee (*Litchi chinensis* Sonn.) peel flour: Effects on hepatoprotection and dyslipidemia induced by a hypercholesterolemic diet. An. Acad. Bras. Cienc..

[14] Huang F., Zhang R., Yi Y., Tang X., Zhang M., Su D., Deng Y., Wei Z. (2014). Comparison of physicochemical properties and immunomodulatory activity of polysaccharides from fresh and dried litchi pulp. Molecules.

[15] Somjai C., Siriwoharn T., Kulprachakarn K., Chaipoot S., Phongphisutthinant R., Chaiyana W., Wiriyacharee P. (2022). Effect of drying process and long-term storage on characterization of Longan pulps and their biological aspects: Antioxidant and cholinesterase inhibition activities. LWT.

[16] Chaipoot S., Wiriyacharee P., Phongphisutthinant R., Buadoktoom S., Srisuwun A., Somjai C., Srinuanpan S. (2023). Changes in physicochemical characteristics and antioxidant activities of dried shiitake mushroom in dry-moist-heat aging process. Foods.

[17] Phongphisutthinant R., Wiriyacharee P., Boonyapranai K., Ounjaijean S., Taya S., Pitchakarn P., Pathomrungsiyounggul P., Utarat P., Wongwatcharayothin W., Somjai C. (2024). Effect of Conventional Humid–Dry Heating through the Maillard Reaction on Chemical Changes and Enhancement of In Vitro Bioactivities from Soy Protein Isolate Hydrolysate–Yeast Cell Extract Conjugates. Foods.

[18] Chen K., Yang Q., Hong H., Feng L., Liu J., Luo Y. (2020). Physicochemical and functional properties of Maillard reaction products derived from cod (*Gadus morhua* L.) skin collagen peptides and xylose. Food Chem..

[19] Liu J., Yong H., Yao X., Hu H., Yun D., Xiao L. (2019). Recent advances in phenolic-protein conjugates: Synthesis, characterization, biological activities and potential applications. RSC Adv..

[20] Magiera A., Czerwińska M.E., Owczarek A., Marchelak A., Granica S., Olszewska M.A. (2022). Polyphenols and Maillard Reaction Products in Dried Prunus spinosa Fruits: Quality Aspects and Contribution to Anti-Inflammatory and Antioxidant Activity in Human Immune Cells Ex Vivo. Molecules.

[21] Ounjaijean S., Chaipoot S., Phongphisutthinant R., Kanthakat G., Taya S., Pathomrungsiyounggul P., Wiriyacharee P., Boonyapranai K. (2024). Evaluation of Prebiotic and Health-Promoting Functions of Honeybee Brood Biopeptides and Their Maillard Reaction Conjugates. Foods.

[22] Somjai C., Siriwoharn T., Kulprachakarn K., Chaipoot S., Phongphisutthinant R., Wiriyacharee P. (2021). Utilization of Maillard reaction in moist-dry-heating system to enhance physicochemical and antioxidative properties of dried whole longan fruit. Heliyon.

[23] Hsu T.Y., Yang K.M., Chiang Y.C., Lin L.Y., Chiang P.Y. (2024). The Browning Properties, Antioxidant Activity, and α-Glucosidase Inhibitory Improvement of Aged Oranges (*Citrus sinensis*). Foods.

[24] Nakagawa K., Maeda H., Yamaya Y., Tonosaki Y. (2020). Maillard Reaction Intermediates and Related Phytochemicals in Black Garlic Determined by EPR and HPLC Analyses. Molecules.

[25] An K., Wu J., Xiao H., Hu T., Yu Y., Yang W., Xiao G., Xu Y. (2022). Effect of various drying methods on the physicochemical characterizations, antioxidant activities and hypoglycemic activities of lychee (*Litchi chinensis* Sonn.) pulp polysaccharides. Int. J. Biol. Macromol..

[26] Zuo X., Wang J., Li Y., Zhang J., Wu Z., Jin P., Cao S., Zheng Y. (2025). Recent advances in high relative humidity strategy for preservation of postharvest fruits and vegetables: A comprehensive review. Food Chem..

[27] Duan X., Huang L., Wang M., Qiao F., Fang C. (2015). Effect of Microwave and Temperature on Quality. J. Food Process. Preserv..

[28] Mahayothee B., Udomkun P., Nagle M., Haewsungcharoen M., Janjai S., Müller J. (2009). Effects of pretreatments on colour alterations of litchi during drying and storage. Eur. Food Res. Technol..

[29] AOAC (2010). Official Methods of Analysis of Association of Official Analytical Chemists.

[30] Zhang R., Zeng Q., Deng Y., Zhang M., Wei Z., Zhang Y., Tang X. (2013). Phenolic profiles and antioxidant activity of litchi pulp of different cultivars cultivated in Southern China. Food Chem..

[31] Gandhi Y.S., Bankar V.H., Vishwakarma R.P., Satpute S.R., Upkare M. (2017). Reducing Sugar Determination of Jaggery by Classical Lane and Eynon Method & 3, 5-Dinitrosalicylic Acid Method. Imp. J. Interdiscip. Res..

[32] Cao X., Islam M.N., Zhong S., Pan X., Song M., Shang F., Nie H., Xu W., Duan Z. (2020). Drying kinetics, antioxidants, and physicochemical properties of litchi fruits by ultrasound-assisted hot air-drying. J. Food Biochem..

[33] Zhu Z., Zhang Y., Wang W., Sun S., Wang J., Li X., Dai F., Jiang Y. (2022). Changes in Physicochemical Properties, Volatile Profiles, and Antioxidant Activities of Black Apple During High-Temperature Fermentation Processing. Front. Nutr..

[34] Pai Y., Pai K.D., Kini M.V. (2022). Experimental investigations on the moisture absorption and mechanical behaviour of basalt-aramid/epoxy hybrid interply composites under different ageing environments. Cogent Eng..

[35] Asikin Y., Nakaza Y., Maeda G., Kaneda H., Takara K., Wada K. (2023). Evaporation Temperature Alters Physicochemical Characteristics and Volatile Maillard Reaction Products of Non-Centrifugal Cane Sugar (NCS): Comparison of Polyethylene Membrane and Retronasal Aroma Simulator Techniques for the Extraction of Volatile Organic Compounds in NCS. Appl. Sci..

[36] Su D., Wang Z., Dong L., Huang F., Zhang R., Jia X., Wu G., Zhang M. (2019). Impact of thermal processing and storage temperature on the phenolic profile and antioxidant activity of different varieties of lychee juice. LWT.

[37] Shu B., Wang J., Wu G., Cao X., Huang F., Dong L., Zhang R., Liu H., Su D. (2022). Newly generated and increased bound phenolic in lychee pulp during heat-pump drying detected by UPLC-ESI-triple-TOF-MS/MS. J. Sci. Food Agric..

[38] Zhu Z., Zhang Y., Wang W., Huang Z., Wang J., Li X., Sun S. (2021). Structural characterisation and antioxidant activity of melanoidins from high-temperature fermented apple. Int. J. Food Sci. Technol..

[39] Stephenus F., Benjamin M.A.Z., Anuar A., Awang M.A. (2023). Effect of Temperatures on Drying Kinetics, Extraction Yield, Phenolics, Flavonoids, and Antioxidant Activity of *Phaleria macrocarpa* (Scheff.) Boerl. (Mahkota Dewa) Fruits. Foods.

[40] Shi J., Yu J., Pohorly J., Young J.C., Bryan M., Wu Y., Canada A. (2003). Optimization of the extraction of polyphenols from grape seed meal by aqueous ethanol solution. J. Food Agric. Environ..

[41] Che Sulaiman I.S., Basri M., Fard Masoumi H.R., Chee W.J., Ashari S.E., Ismail M. (2017). Effects of temperature, time, and solvent ratio on the extraction of phenolic compounds and the anti-radical activity of Clinacanthus nutans Lindau leaves by response surface methodology. Chem. Cent. J..

[42] Aoudeh E., Oz E., Kelebek H., Brennan C., Ahmad N., Elobeid T., Oz F. (2023). Black garlic production: The influence of ageing temperature and duration on some physicochemical and antioxidant properties, and sugar content. Int. J. Food Sci. Technol..

[43] Shin J.-H., Kang M.-J., Lee B.H., Kang D. (2024). Effect of Temperature Conditions on the Physicochemical Quality of Aged Black Garlic. Foods.

[44] Laroque D., Inisan C., Berger C., Vouland E., Dufossé L., Guérard F. (2008). Kinetic study on the Maillard reaction. Consideration of sugar reactivity. Food Chem..

[45] Alonso-Riaño P., Illera A., Benito-Román Ó., Melgosa R., Bermejo-López A., Beltrán S., Sanz M. (2024). Degradation kinetics of sugars (glucose and xylose), amino acids (proline and aspartic acid) and their binary mixtures in subcritical water: Effect of Maillard reaction. Food Chem..

[46] Hashimoto K., Niina T., Kobayashi T., Adachi S., Watanabe Y. (2024). Isomerization and epimerization of fructose in phosphate buffer under subcritical water conditions. Carbohydr. Res..

[47] Teodorowicz M., Van Neerven J., Savelkoul H. (2017). Food Processing: The Influence of the Maillard Reaction on Immunogenicity and Allergenicity of Food Proteins. Nutrients.

[48] Chaipoot S., Wiriyacharee P., Pathomrungsiyounggul P., Kanthakat G., Somjai C., Boonyapranai K., Srinuanpan S., Wongwatcharayothin W., Phongphisutthinant R. (2025). Antioxidant Activity and Chemical Alterations of Honeybee Brood Bio-Peptides Interacting with Honey Under Moist-Dried Thermal Aging. Antioxidants.

[49] Réblová Z. (2012). Effect of temperature on the antioxidant activity of phenolic acids. Czech J. Food Sci..

[50] Wang Z., Wu G., Shu B., Huang F., Dong L., Zhang R., Su D. (2020). Comparison of the phenolic profiles and physicochemical properties of different varieties of thermally processed canned lychee pulp. RSC Adv..

